# Prevention of Bleomycin-Induced Pulmonary Inflammation and Fibrosis in Mice by Paeonol

**DOI:** 10.3389/fphys.2017.00193

**Published:** 2017-03-31

**Authors:** Meng-Han Liu, An-Hsuan Lin, Hsin-Kuo Ko, Diahn-Warng Perng, Tzong-Shyuan Lee, Yu Ru Kou

**Affiliations:** ^1^Department of Physiology, School of Medicine, National Yang-Ming UniversityTaipei, Taiwan; ^2^Department of Chest Medicine, Taipei Veterans General HospitalTaipei, Taiwan

**Keywords:** paeonol, bleomycin, pulmonary inflammation, pulmonary fibrosis, collagen, α-smooth muscle actin, mice, lung fibroblasts

## Abstract

Pulmonary fibrosis is a severe and progressive disease that is characterized by an abnormal deposition of extracellular matrix, such as collagens. The pathogenesis of this disease may be initiated by oxidative damage of lung epithelial cells by fibrogenic stimuli, leading to lung inflammation, which in turn promotes various lung fibrotic responses. The profibrogenic effect of transforming growth factor-β1 (TGF-β1) on lung fibroblasts is crucial for the pathogenesis of this disease. Paeonol, the main phenolic compound present in the Chinese herb *Paeonia suffruticosa*, has antioxidant and anti-inflammatory properties. However, whether paeonol has therapeutic effects against pulmonary fibrosis remains unclear. Using a murine model, we showed that 21 days after the insult, intratracheal bleomycin caused pulmonary inflammation and fibrosis, as evidenced by lung histopathological manifestations and increase in various indices. The inflammatory indices included an increase in total cell count, differential cell count, and total protein concentration in bronchoalveolar lavage fluid. The fibrotic indices included an increase in lung levels of TGF-β1, total collagen, type 1α1 collagen (COL1A1), and α-smooth muscle actin (α-SMA; a marker of myofibroblasts). Bleomycin also was found to cause an increase in oxidative stress as reflected by increased levels of malondialdehyde and 4-hydroxynonenal in the lungs. Importantly, all these pathophysiological events were suppressed by daily treatment with paeonol. Using human lung fibroblasts, we further demonstrated that exposure of human lung fibroblasts to TGF-β1 increased productions of α-SMA and COL1A1, both of which were inhibited by inhibitors of Jun N-terminal kinase (JNK), p38, and Smad3. JNK and p38 are two subfamily members of mitogen-activated protein kinases (MAPKs), whereas Smad3 is a transcription factor. TGF-β1 exposure also increased the phosphorylation of JNK, p38, and Smad3 prior to the induction of α-SMA and COL1A1. Notably, all these TGF-β1-induced cellular events were suppressed by paeonol treatment. Our findings suggest that paeonol has antioxidant, anti-inflammatory, and anti-fibrotic functions against bleomycin-induced pulmonary fibrosis in mice. The beneficial effect of paeonol may be, at least in part, mediated through the inhibition of the MAPKs/Smad3 signaling.

## Introduction

Pulmonary fibrosis is a severe and progressive disease associated with considerable morbidity and mortality in humans (Hutchinson et al., 2015; Raghu et al., 2015). It is characterized by destruction of alveolar structures with an abnormal deposition of extracellular matrix, such as collagen (Wilson and Wynn, 2009; Wuyts et al., 2013). The etiology of pulmonary fibrosis varies, and this disease has an array of triggers, including chemicals, radiation, fibrogenic environmental toxins, or other unknown factors (Wilson and Wynn, 2009). The development of pulmonary fibrosis involves sequential events, including injury, inflammation, and repair, and dysregulation at one or more of these events is essential for its pathogenesis (Wilson and Wynn, 2009; Wuyts et al., 2013). Oxidative stress may be one factor that participates in the initiation and progression of these events (Cheresh et al., 2013). Thus, the pathogenesis of this disease may be initiated by damage of lung epithelial cells by fibrogenic stimuli, thereby leading to lung inflammation, which is regulated by various types of cells and cytokines (Cheresh et al., 2013; Wuyts et al., 2013). To this end, transforming growth factor-β1 (TGF-β1) is the most potent profibrogenic cytokine (Wuyts et al., 2013). TGF-β1 can directly stimulate lung fibroblasts to secrete collagens or induce transformation of fibroblasts to myofibroblasts that express α-smooth muscle actin (α-SMA); both are key events in the pathogenesis of pulmonary fibrosis (Horowitz et al., 2007; Tseng et al., 2013; Kasabova et al., 2014; Deng et al., 2015). These TGF-β1-mediated events are known to be mediated through distinct signaling pathways, such as mitogen-activated protein kinases (MAPKs) and Smad 3 (Horowitz et al., 2007; Tseng et al., 2013; Kasabova et al., 2014; Deng et al., 2015; Sato et al., 2016). The pathogenesis of pulmonary fibrosis has been widely studied, but the search for therapeutic interventions remains challenging (Wuyts et al., 2013).

Paeonol (2′-hydroxy-4′-methoxyacetophenone), the main phenolic compound of the radix of the Chinese herb *Paeonia suffruticosa* (*Cortex Moutan*), has been used as a traditional herbal medicine for thousands of years (Zhang et al., 1996; Matsuda et al., 2001). Paeonol has been demonstrated to have antioxidant activity when used in *ex vitro* (Zhang et al., 1999; Matsuda et al., 2001; Jin et al., 2016), *in vitro* (Tseng et al., 2012; Liu et al., 2014; Ping et al., 2014), and *in vivo* preparations (Hsieh et al., 2006; Liu et al., 2014; Ding et al., 2016; Jin et al., 2016). Paeonol also has anti-inflammatory actions in a number of animal models of diseases other than pulmonary fibrosis (Chou, 2003; Du et al., 2010; Fu et al., 2012; Zhao et al., 2013; Chen et al., 2014; Liu et al., 2014; Ding et al., 2016; Zong et al., 2017) and in various cell types in response to stimuli (Nizamutdinova et al., 2007; Chae et al., 2009; Du et al., 2010; Himaya et al., 2012; Tseng et al., 2012; Kong et al., 2013; Liu et al., 2014; He et al., 2016; Jin et al., 2016). Thus, the antioxidant and anti-inflammatory properties of paeonol make it a potential drug for the therapy of pulmonary fibrosis. However, this potential remains to be proven.

We aimed to investigate the therapeutic effects of paeonol on pulmonary inflammation and fibrosis *in vivo* and to determine any therapeutic mechanism underlying the beneficial effects of paeonol *in vitro*. We used a murine model with bleomycin insult (Chua et al., 2005; Della Latta et al., 2015) to assess the inhibitory effects of paeonol on various indices of oxidative stress, inflammation, and fibrosis in the lungs. Additionally, we used an established *in vitro* model of lung fibroblasts (Tseng et al., 2013; Kasabova et al., 2014) to determine the suppressive effects of paeonol on the TGF-β1-mediated activation of the MAPKs/Smad3 signaling pathway and on the increase in fibrotic responses.

## Methods

### Reagents

Antibodies (Abs) to measure α-SMA, type 1α1 collagen (COL1A1), Jun N-terminal kinase (JNK), p38, Smad3, phospho-JNK, phospho-p38, and phospho-Smad3 were obtained from Santa Cruz Biotechnology (Santa Cruz, CA, USA). Sircol Collagen Assay kit was purchased from Biocolor Ltd. (Carrickfergus, UK). ELISA kit used to measure TGF-β1 was purchased from Enzo Life Sciences (Farmingdale, NY, USA). Rabbit antibody against 4-hydroxynonenal (4-HNE) was purchased from Abcam (Cambridge, MA, USA). Mouse antibody against α-tubulin, paeonol (purity > 99%, HPLC), N-acetyl-cysteine, SIS3, Masson's trichrome stain kit, and malondialdehyde (MDA) kit were purchased from Sigma-Aldrich (St. Louis, MO, USA). SB203580, SP600125, and PD98059 were obtained from Calbiochem (San Diego, CA, USA). Bleomycin was purchased from Nippon Kayaku (Tokyo, Japan).

### Murine model of pulmonary fibrosis and paeonol treatment

All animal experiments were approved by the Animal Care and Use Committee of the National Yang-Ming University. Eight-week old C57BL/6J mice (National Laboratory Animal Center, Taipei, Taiwan) were randomly divided into four experimental groups: Control (PBS, the vehicle of bleomycin) group, PBS+paeonol group, bleomycin+saline group (saline, the vehicle of paeonol), and bleomycin+paeonol group. To induce pulmonary fibrosis, mice were treated with a single sublethal dose of bleomycin (3 mg/kg) via intratracheal infusion. For the PBS groups, the same protocol was conducted, but instead of bleomycin, the mice received the same volume of intratracheal PBS. For the therapeutic treatment, mice received daily treatment with paeonol (10 mg/kg) or saline by gastric gavage for 21 days. Animals in each group were euthanized 21 days after intratracheal bleomycin administration. The dose of daily treatment with paeonol was adopted from our recent study (Liu et al., 2014). The dose of bleomycin and protocol of the induction of pulmonary fibrosis were adopted from a study reported previously (Izbicki et al., 2002).

### Preparation of bronchoalveolar lavage fluid (BALF) and lung tissues

At the end of each experiment, the mice were euthanized with CO_2_, and a middle thoracotomy was performed. The left lung was ligated, and the right lung was lavaged four times with 0.4 ml warm PBS containing a complete protease inhibitor cocktail (Roche Diagnostics, Mannheim, Germany). The BALF samples were then centrifuged at 350 × g for 5 min at 4°C, and the supernatant of the first lavage fluid was stored at −80°C for analysis of total protein using Bio-Rad protein assay reagent (Bio-Rad Laboratories, Hercules, CA, USA). The cell pellets of the BALF samples were re-suspended in PBS for cell counting. Furthermore, the right lung was then stored at −80°C for subsequent analysis. The left lung was fixed with 4% paraformaldehyde and embedded in paraffin.

### Histological assessment

Formalin-fixed, paraffin-embedded tissue blocks were cut into 8-μm sections. Sections were deparaffinized, rehydrated, and then subjected to hematoxylin and eosin (H&E) staining and Masson's trichrome staining to investigate levels of lung inflammation and collagen deposition. These sections were viewed under a microscope (Motic TYPE 102M, Xiamen, China).

### Measurement of indices of pulmonary fibrosis

The concentrations of TGF-β1 and the total collagen in the lung tissue samples were measured using ELISA kits and the Sircol Collagen Assay kit, respectively, according to the manufacturer's instructions. In addition, the lung tissue samples were lysed with phosphate buffered saline containing 1% Triton X-100, 0.1% SDS, 0.5% sodium deoxycholate, 1 μg/mL leupeptin, 10 μg/mL aprotinin, 1 mM PMSF, Tyr phosphatase cocktail I and Ser/Thr phosphatase cocktail II on ice. Tissue extracts underwent centrifugation at 12,000 × g for 5 min at 4°C, and supernatants were collected as tissue lysates. The tissue lysates were used to measure the level of α-SMA and COL1A1 by Western blot analysis.

### Measurement of indices of oxidative stress

Levels of MDA and 4-HNE in the lung tissue samples were measured by assay kits and Western blot analysis, respectively. The levels of these two products of lipid peroxidation were used to reflect the levels of oxidative stress (Li et al., 2015; Yoon et al., 2016).

### Cell culture

Human fetal lung fibroblasts (HFL-1) were purchased from Taiwan Medical Cell and Microbial Resources (Hsinchu, Taiwan). Lung fibroblasts were cultured in F12K medium containing 10% fetal bovine serum (FBS), 1 × low serum growth supplement, 100 U/mL penicillin, 100 μg/mL streptomycin, and 0.25 μg/mL amphotericin B (Biological Industries, Kibbutz Beit Haemek, Israel) at 37°C in an incubator supplied with 5% CO_2_.When the cells reached 80% confluence, they were stimulated by human recombinant TGF-β1 (0–5 ng/ml) with or without paeonol treatment (0–0.4 mM). The concentration of TGF-β1 (Kasabova et al., 2014) and paeonol (Liu et al., 2014) were adopted from studies reported previously.

### Western blot analysis

The cell lysates were prepared using cell lysis buffer (Cell Signaling, Beverly, MA, USA). Aliquots of cell lysates or tissue lysates were separated by 8–12% SDS-PAGE and then transblotted onto Immobilon™-P membrane (Millipore). After being blocked with 5% skim milk, the blots were incubated with various primary antibodies and then appropriate secondary antibodies. The specific protein bands were detected using an enhanced chemiluminescence kit (Perkin-Elmer), which was followed by the quantification using ImageQuant 5.2 software (Healthcare Bio-Sciences, Philadelphia, PA, USA).

### Statistical analysis

The results are presented as mean ± SEM. Statistical evaluations involved one-way ANOVA followed by Dunnett's test or Fisher's least significant difference procedure for multiple comparisons as appropriate. Differences were considered statistically significant at *p* < 0.05.

## Results

### Effect of paeonol on pathological manifestations in the mouse lung

**Figure 1:**
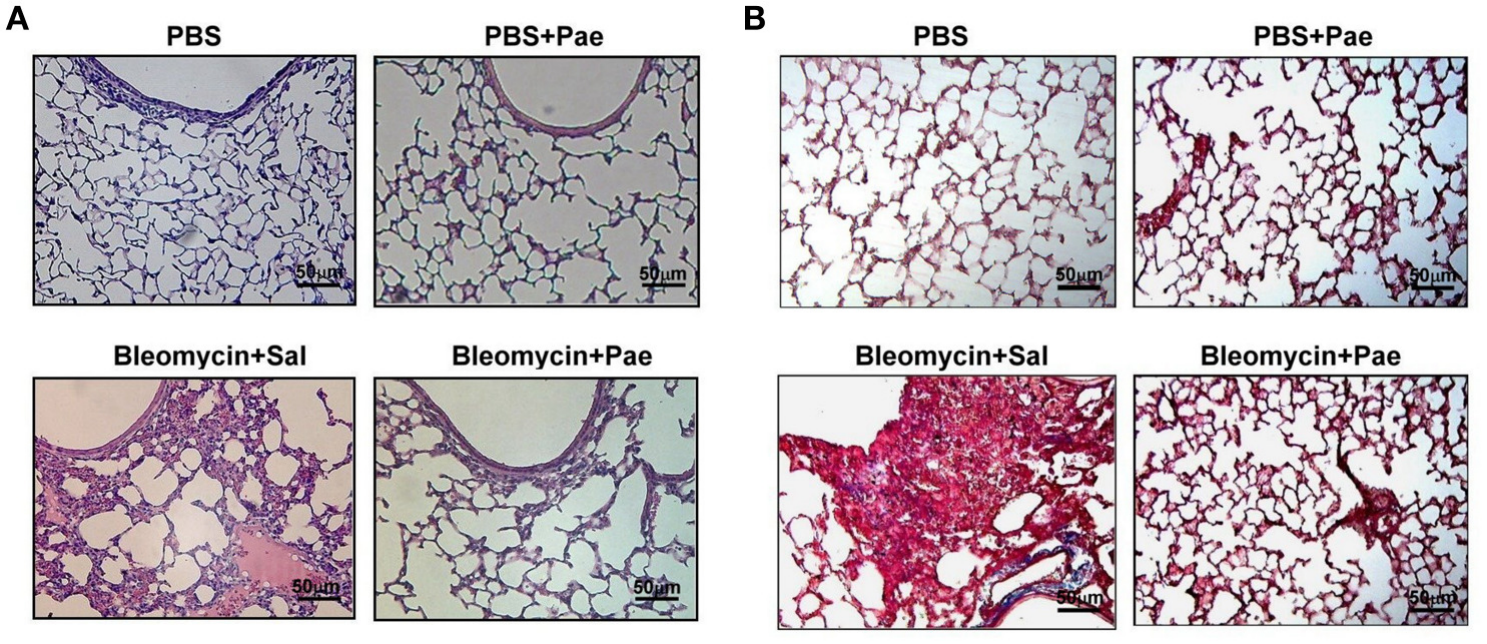
**Representative images of H&E stained (A)** and Masson's trichrome **(B)** lung sections obtained from mice of 4 study groups. Mice were intratracheally infused with either PBS or a single sublethal dose of bleomycin (3 mg/kg). Mice also received daily treatment with paeonol (10 mg/kg; Pae) or saline (Sal.) by gastric gavage for 21 days. Lung tissues were obtained 21 days after bleomycin treatment. The magnification is 200X for each panel. Note that bleomycin resulted in extensive infiltration of inflammatory cells **(A)** and collagen deposition, as indicated by the blue color **(B)**. These signs of pulmonary inflammation and fibrosis were markedly alleviated by paeonol treatment.

Twenty one days after bleomycin administration, mice showed both inflammation and fibrosis in the lungs. When compared to the PBS control mice, bleomycin produced pathological manifestations, including increased number of pulmonary interstitial cells (e.g., inflammatory cells and fibroblasts), thickening of the alveolar walls, focal regions of damaged alveolar structure, granulomatous lesions in the alveolar space, and distorted pulmonary architecture, as evidenced by the histological evaluation of the H&E stained lung sections (Figure 1A). Additionally, mice challenged with bleomycin showed notable deposition of collagen in the pulmonary interstitium, as indicated by Masson's trichrome staining (Figure 1B). Importantly, all these pathological manifestations were largely alleviated in the bleomycin-insulted mice that underwent paeonol treatment (Figures 1A,B). These pathological manifestations were not found in the PBS-insulted mice that underwent paeonol treatment (Figures 1A,B).

### Effect of paeonol on indices of inflammation, fibrosis, and oxidative stress in the mouse lung

**Figure 2:**
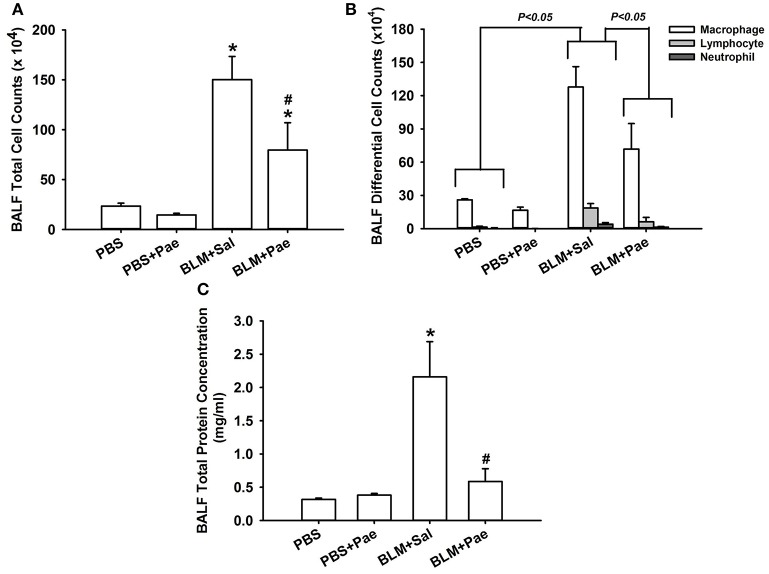
**Treatment with paeonol (Pae) alleviates bleomycin (BLM)-induced increase in indices of pulmonary inflammation in mice**. The indices measured were total cell count **(A)**, differential cell count **(B)**, and total protein concentration **(C)** in bronchoalveolar lavage fluid (BALF) sampled from mice of 4 study groups. Data in each group are mean ± SEM from 6 mice. ^*^p < 0.05 vs. the PBS group; ^#^*p* < 0.05 vs. the BLM group. See legend of Figure 1 for treatments of 4 study groups. See the legend of Figure 1 for detailed information on each study group.

**Figure 3:**
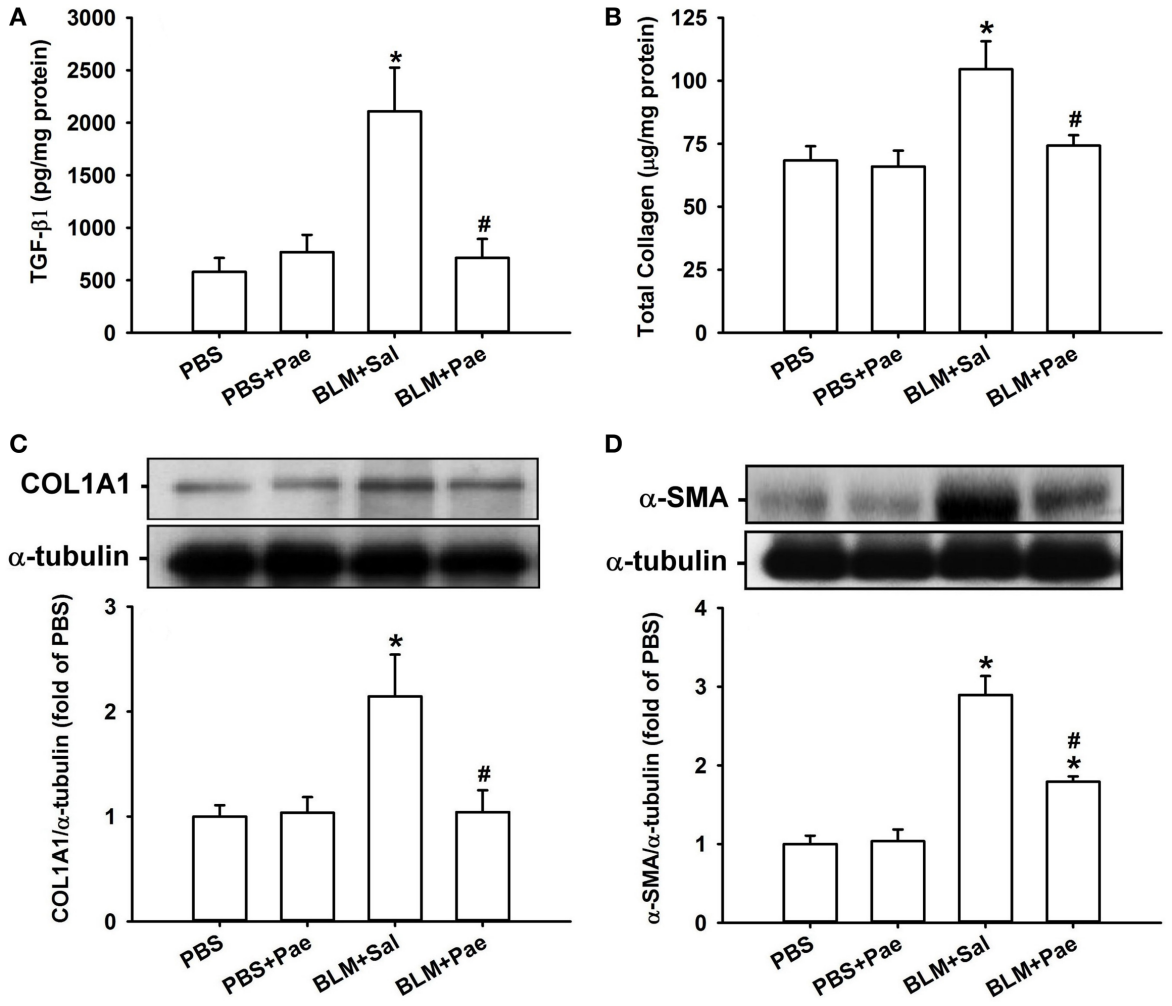
**Treatment with paeonol (Pae) alleviates bleomycin (BLM)-induced increases in indices of pulmonary fibrosis in mice**. The indices measured were levels of transforming growth factor-β1 (**A**; TGF-β1), total collagen **(B)**, type 1α1 collagen (**C**; COL1A1), and α-smooth muscle actin (**D**; α-SMA) in lung tissues sampled from mice of 4 study groups. Data in **(A,B)** were measured by ELISA. Data in **(C,D)** were measured by Western blot analysis. Data in each group are mean ± SEM from 6 mice. ^*^p < 0.05 vs. the PBS group; ^#^*p* < 0.05 vs. the BLM group.

**Figure 4:**
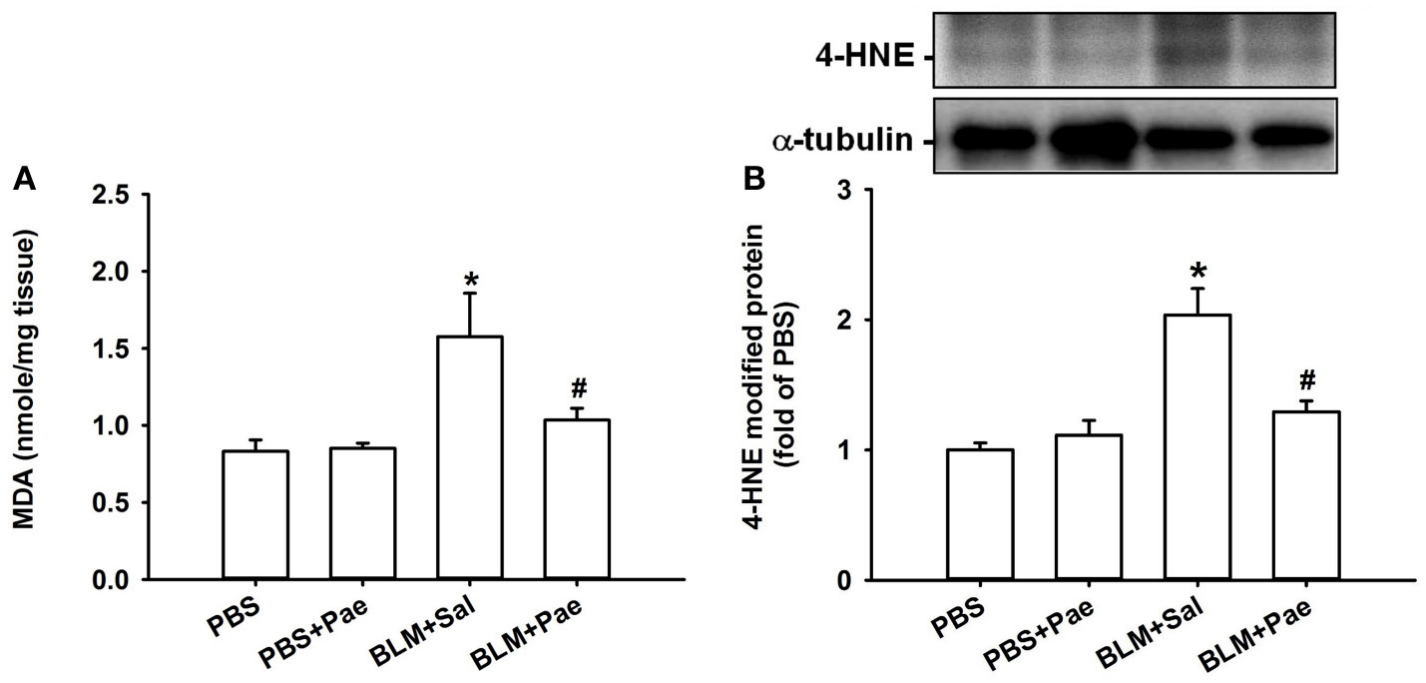
**Treatment with paeonol (Pae) alleviates bleomycin (BLM)-induced increase in indices of pulmonary oxidative stress in mice**. The indices measured were levels of malondialdehyde (MDA, **A**) and 4-hydroxynonenal modified proteins (4-HNE, **B**) in lung tissues sampled from mice belonging to four study groups. Data in **(A)** were measured by an assay kit, whereas data in **(B)** were measured by Western blot analysis. Data in each group are mean ± SEM from 6 mice. ^*^p < 0.05 vs. the PBS group; ^#^*p* < 0.05 vs. the BLM group.

Compared with the PBS control mice, mice with intratracheal bleomycin showed an increase in total cell count (Figure 2A), differential cell count (Figure 2B), and total protein concentrations in BALF (Figure 2C), all of which are indices of pulmonary inflammation. Additionally, bleomycin-insulted mice showed increased lung levels of TGF-β1 (Figure 3A) and total collagen concentration (Figure 3B), as measured by ELISA, and increased lung expression of COL1A1 (Figure 3C) and α-SMA (Figure 3D), as assessed by Sircol Collagen Assay kit or Western blot analysis; all these are indices of pulmonary fibrosis. Furthermore, bleomycin-insulted mice showed an increase in lung levels of MDA (Figure 4A), as measured by ELISA and 4-HNE (Figure 4B), as assessed by Western blot analysis; all these are indices of pulmonary oxidative stress. Importantly, all these pathophysiological indices were significantly reduced in the bleomycin-insulted mice that underwent paeonol treatment (Figures 2–4). These pathophysiological indices did not increase in mice subjected to PBS insult and underwent paeonol treatment (Figures 2–4).

### Effect of paeonol on the TGF-β1-mediated induction of α-SMA and type 1α1 collagen in lung fibroblasts

**Figure 5:**
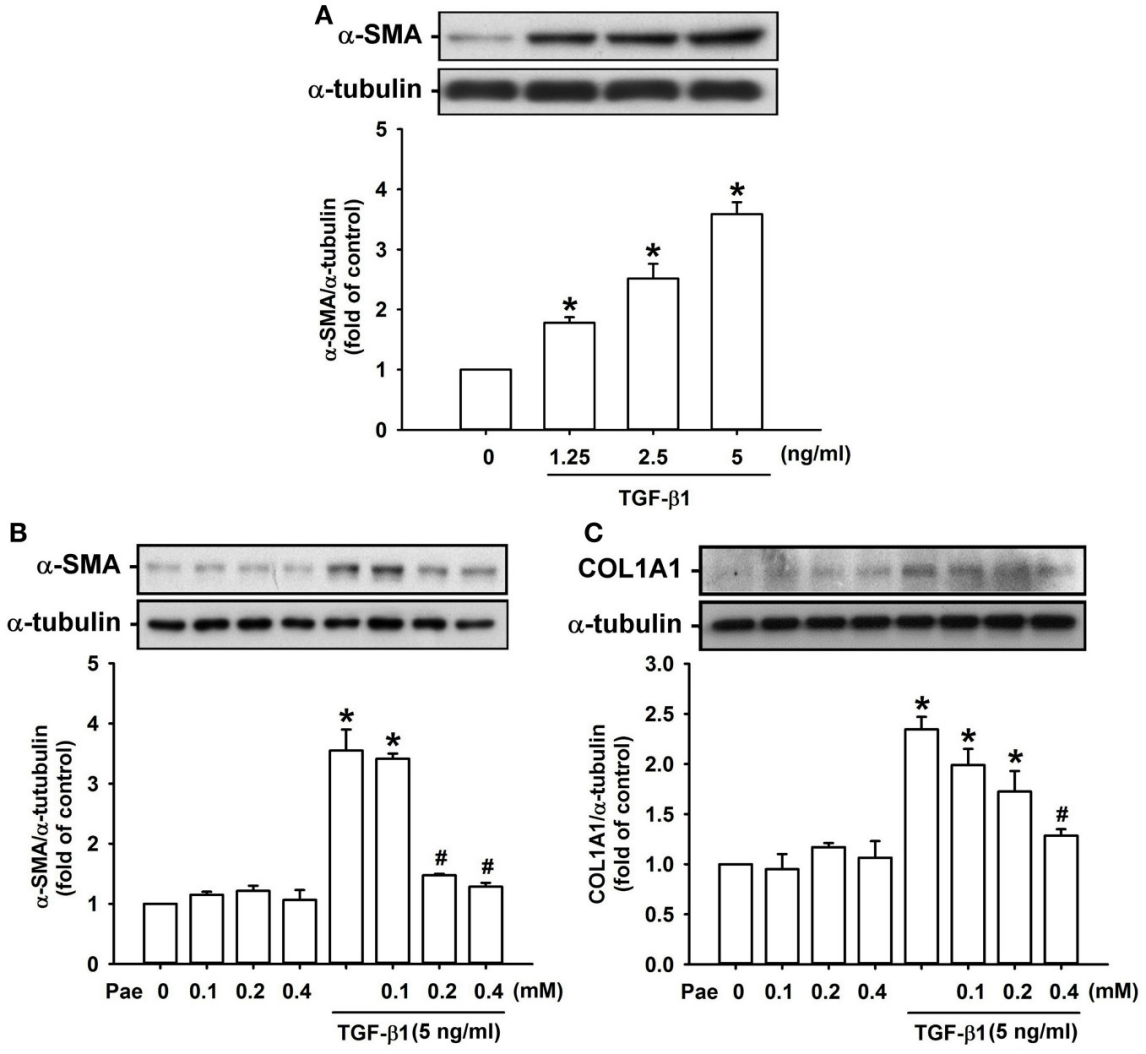
**Treatment with paeonol (Pae) inhibits TGF-β1-induced increases in expression of α-smooth muscle actin (α-SMA) and type 1α1 collagen (COL1A1) in human lung fibroblasts**. In **(A)**, stimulatory effects of different concentrations of TGF-β1 (0–5 ng/ml) on α-SMA were studied. In **(B,C)**, the suppressive effects of Pae (0–0.4 mM) on TGF-β1 (5 ng/ml)-induced α-SMA and COL1A1 were studied. Protein samples were harvested 24 h after treatments and data were measured by Western blot analysis. Data in each group are mean ± SEM from 4 independent experiments. ^*^p < 0.05 vs. the control group; ^#^*p* < 0.05 vs. the TGF-β1 group without Pae treatment.

We used TGF-β1-stimulated human lung fibroblasts as an *in vitro* model to study the therapeutic mechanism of paeonol. Exposure of lung fibroblasts to 0–5 ng/ml TGF-β1 for 24 h induced a concentration-dependent increase in expression of α-SMA (Figure 5A). Pretreatment with various concentrations of paeonol (0, 0.1, 0.2, and 0.4 mM) concentration-dependently attenuated the induction of α-SMA (Figure 5B) and COL1A1 (Figure 5C) by TGF-β1, whereas pretreatment with paeonol in lung fibroblasts without TGF-β1 stimulation failed to alter the expression of α-SMA and COL1A1 (Figures 5B,C). Based on the abovementioned result, we used 5 ng/ml TGF-β1 and 0.4 mM paeonol as the standard stimulus and treatment, respectively, for the subsequent experiments. Exposure to bleomycin did not alter expression of a-SMA and type COL1A1 in human lung fibroblasts (Supplemental Material).

### Effect of paeonol on the TGF-β1-mediated activation of MAPKs/Smad3 signaling in lung fibroblasts

**Figure 6:**
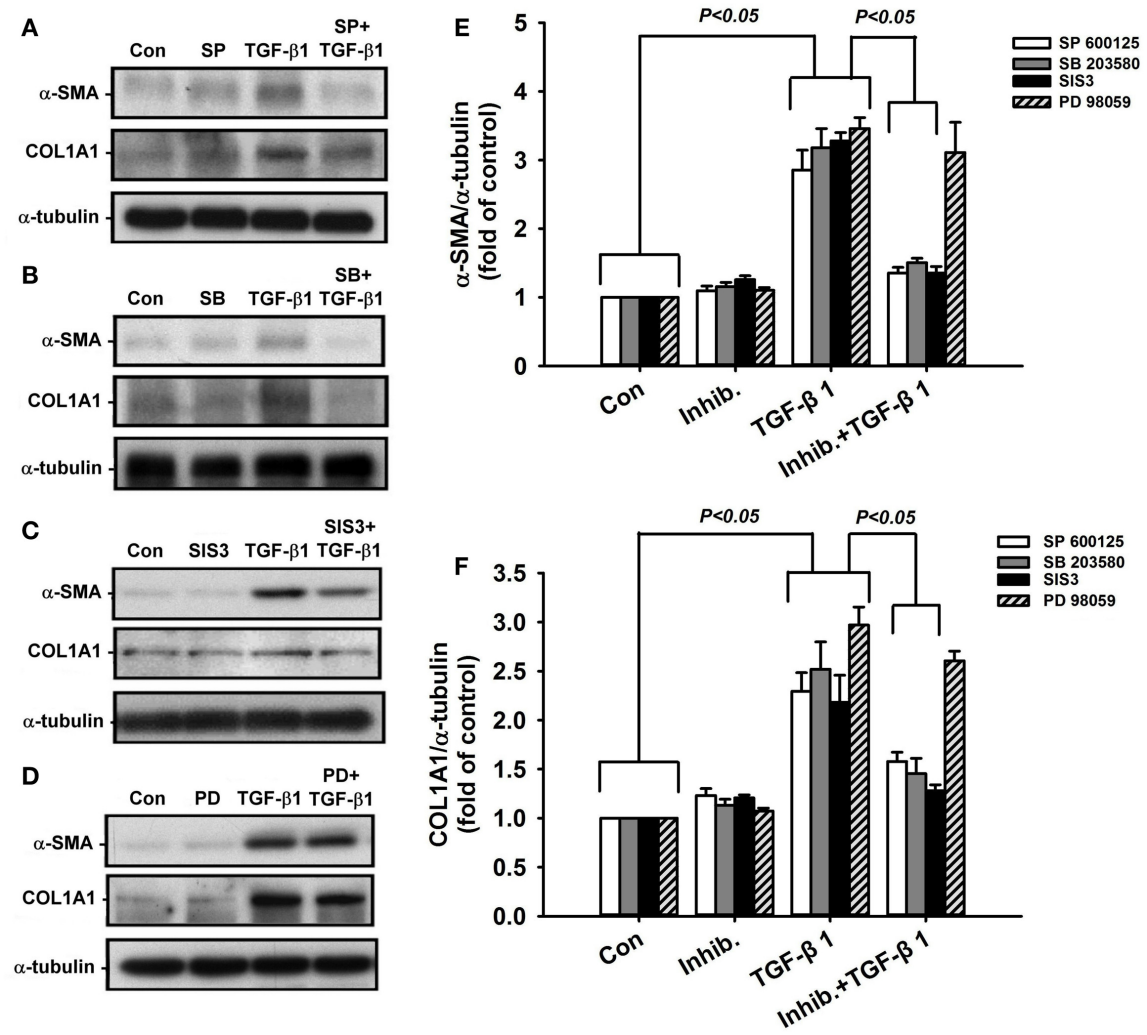
**Suppressive effects of various pharmacological inhibitors on TGF-β1-induced increases in expression of α-smooth muscle actin (α-SMA) and type 1α1 collagen (COL1A1) in human lung fibroblasts**. The inhibitors (Inhib.) used were JNK inhibitor (**A**; SP600125, 10 μM), p38 inhibitor (**B**; SB203580, 10 μM), Smad3 inhibitor (**C**; SIS3, 3 μM), and ERK inhibitor (**D**; PD98059, 10 μM). Group data of expression of α-SMA and COL1A1 are shown in **(E,F)**. Protein samples were harvested 24 h after treatments and data were measured by Western blot analysis. Data in each group are mean ± SEM from 4 independent experiments. Note that only PD98059 had no significant effect (*p* > 0.05) on TGF-β1-induced increase in expression of α-SMA and COL1A1.

**Figure 7:**
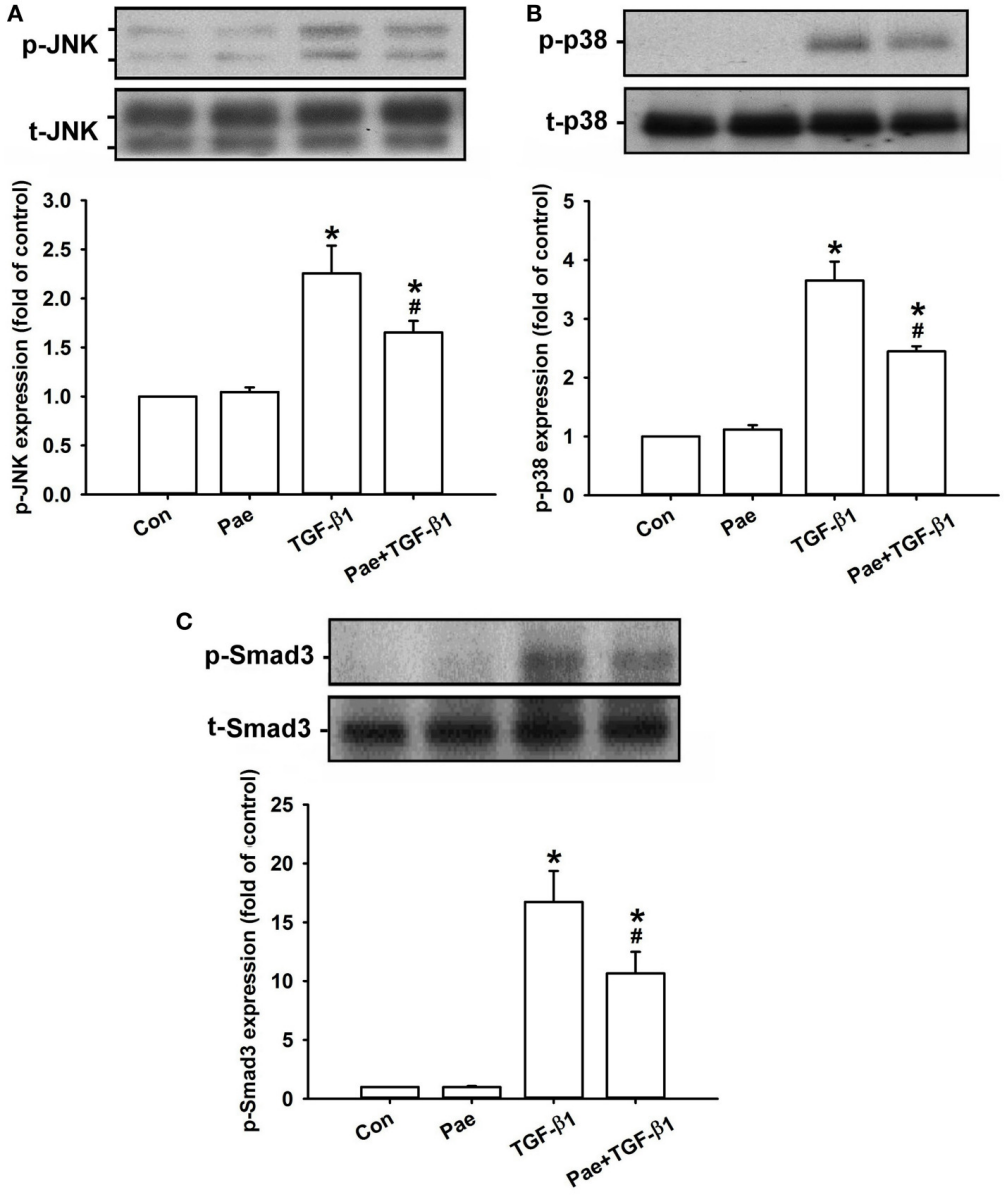
**Treatment with paeonol (Pae) inhibits TGF-β1-induced increases in phosphorylation of JNK (A)**, p38 **(B)**, and Smad3 **(C)** in human lung fibroblasts. Pae (0.4 mM) was used to suppress TGF-β1 (5 ng/ml)-induced increases in phosphorylation. Protein samples were harvested 1 h after treatment. Levels of phospho- (p-) and total- (t-) proteins were measured by Western blot analysis. Data in each group are mean ± SEM from 4 independent experiments. ^*^p < 0.05 vs. the control group; ^#^*p* < 0.05 vs. the TGF-β1 group.

Activation of MAPKs and Smad3 is known to be important in inducing fibrotic responses to TGF-β1 in lung fibroblasts (Horowitz et al., 2007; Tseng et al., 2013; Kasabova et al., 2014; Deng et al., 2015). Using various pharmacological inhibitors, we found that exposure of lung fibroblasts to 5 ng/ml TGF-β1 for 24 h produced induction of α-SMA or COL1A1, both of which were inhibited by pretreatment with either a JNK inhibitor (SP600125), a p38 inhibitor (SB203580), or a Smad3 inhibitor (SIS3), but were unaffected by pretreatment with an ERK inhibitor (PD98059) (Figure 6). These results suggest the important roles of JNK and p-38 (two members of MAPKs), and Smad3 (a transcription factor) in these TGF-β1-mediated fibrotic responses. Additionally, we found that, relative to the control group, exposure of lung fibroblasts to 5 ng/ml TGF-β1 for 1 h indeed increased the amount of phosphorylated JNK (Figure 7A), phosphorylated p-38 (Figure 7B), and phosphorylated Smad3 (Figure 7C). Such TGF-β1-induced activation of the MAPKs/Smad3 signaling was significantly attenuated by pretreatment with 0.4 mM paeonol (Figure 7). Pretreatment with paeonol in lung fibroblasts without TGF-β1 stimulation failed to alter the expression of these signaling proteins (Figure 7). Our results thus established the relationship between phosphorylations of MAPKs or Smad3 and α-SMA or COL1A1.

## Discussion

Our *in vivo* study demonstrates clearly that 21 days after the insult, bleomycin caused pulmonary inflammation and fibrosis, as evidenced by lung histopathological manifestations and increase in various indices. The inflammatory indices measured were total cell count, differential cell count, and total protein concentration in BALF. The fibrotic indices measured were lung levels of TGF-β1 (a potent profibrogenic cytokine), total collagen, COL1A1, and α-SMA (a marker of myofibroblasts). Bleomycin also increased oxidative stress, as reflected by increased levels of MDA and 4-HNE in the lungs. All these pathophysiological events were suppressed by daily treatment with paeonol, suggesting that paeonol has antioxidant, anti-inflammatory, and anti-fibrotic functions against bleomycin-induced pulmonary fibrosis in mice. Our *in vitro* study demonstrated that exposure of lung fibroblasts to TGF-β1 increased the production of α-SMA and COL1A1, both of which were inhibited by inhibitors of JNK, p38, and Smad3. The important roles of these signaling proteins gain support from our observations, revealing that TGF-β1 exposure indeed increased their phosphorylated amount prior to the induction of α-SMA and COL1A1. Notably, all these TGF-β1-induced cellular events were suppressed by paeonol treatment, thereby indicating that the beneficial effect of paeonol may be, at least in part, mediated through the inhibition of the MAPKs/Smad3 signaling.

Our study appears to be the first to report that paeonol has antioxidant, anti-inflammatory, and anti-fibrotic activities against bleomycin-induced pulmonary fibrosis. Paeonol has been reported to possesses antioxidant activity against oxidative stress *ex vitro* (Zhang et al., 1999; Matsuda et al., 2001; Jin et al., 2016), *in vivo* (Hsieh et al., 2006; Liu et al., 2014; Ding et al., 2016), and *in vitro* (Tseng et al., 2012; Liu et al., 2014; Ping et al., 2014). Paeonol suppressed the inflammation in the lungs with cigarette smoke exposure (Liu et al., 2014), acetaminophen-induced liver injury (Ding et al., 2016), drug-induced colitis (Jin et al., 2016; Zong et al., 2017), ischemia-reperfusion brain injury (Hsieh et al., 2006), lipopolysaccharide-induced lung injury (Fu et al., 2012; Chen et al., 2014), ovalbumin-induced hyperresponsive airways (Du et al., 2010), atherosclerosis (Zhao et al., 2013), and carrageenan-evoked thermal hyperalgesia (Chou, 2003) in several animal models. Paeonol has been demonstrated to inhibit the inflammatory responses in cigarette smoke-stimulated lung epithelial cells (Liu et al., 2014), in lipopolysaccharide-stimulated macrophages (Chae et al., 2009; Himaya et al., 2012; Chen et al., 2014; Jin et al., 2016), in microglial cells (Himaya et al., 2012; Tseng et al., 2012; He et al., 2016), and in TNF-α-stimulated endothelial cells (Nizamutdinova et al., 2007). Particularly, the anti-inflammatory effect of paeonol *in vitro* appears to be due to inactivation of the signaling pathways involved (Nizamutdinova et al., 2007; Chae et al., 2009; Himaya et al., 2012; Tseng et al., 2012; Chen et al., 2014; Liu et al., 2014; Jin et al., 2016). Only one study reported the therapeutic effect of paeonol on carbon tetrachloride-induced hepatic fibrosis in rats by inhibiting activation of hepatic stellate cells (Kong et al., 2013). Thus, our findings are in good agreement with the abovementioned observations. In this study, we measured *in vivo* responses 21 days after bleomycin administration, a duration that allowed us to observe both inflammatory and fibrotic responses, as reported previously (Izbicki et al., 2002; Della Latta et al., 2015). All these bleomycin-induced pathophysiological events are similar to those described previously (Izbicki et al., 2002; Chua et al., 2005; Li et al., 2015). Particularly, macrophages were the predominant cells in the inflammatory cell infiltration at 21 days after bleomycin insult, a finding that is consistent with those reported previously (Izbicki et al., 2002). This experimental model has been widely used to investigate on the pathogenesis and potential therapies of pulmonary fibrosis (Chua et al., 2005; Della Latta et al., 2015). In this model, bleomycin causes oxidative lung injury and this leads to lung inflammation, which in turn promotes various lung fibrotic responses (Chua et al., 2005; Hecker et al., 2009; Della Latta et al., 2015). Since paeonol was found to effectively reduce the lung oxidative stress in our model, one possible therapeutic mechanism is the suppression of oxidative lung injury, which would alleviate the subsequent inflammatory and fibrotic events. Also, pulmonary responses of oxidative stress and inflammation induced by bleomycin are interrelated and this suggests that suppression of one response may lead to inhibition of the other. The bleomycin-induced lung oxidative stress has been suggested to be a result from activation of NADPH oxidases and/or imbalance between oxidation and anti-oxidation capacity (Cheresh et al., 2013). Additionally, our *in vitro* findings regarding the TGF-β1-induced activation of the MAPKs/Smad3 signaling and productions of α-SMA and COL1A1 in lung fibroblasts are in agreement with observations reported by other investigators (Horowitz et al., 2007: Kasabova et al., 2014; Tseng et al., 2013; Deng et al., 2015). Previous investigations have shown that the TGF-β1-induced activation of p38 and Smad3 and, fibrotic responses in fibroblasts are mediated through the generation of reactive oxygen species (Alili et al., 2014; Sato et al., 2016). Thus, the same antioxidant function of paeonol can also explain our *in vitro* findings. The other possible therapeutic mechanism is that paeonol directly suppresses lung inflammation and fibrosis by inhibiting related signaling pathways. In fact, the biological activities of paeonol are known to be largely related to its ability to modulate associated signaling pathways (Tang et al., 2016). In our *in vivo* study, we speculate that the target cells for paeonol may include all types of cells that participate in the development of lung fibrosis, such as lung epithelial cells, endothelial cells, fibroblasts, and myofibroblasts.

In our *in vivo* study, the dose of paeonol (10 mg/kg) used is only one tenth of the dose used in previous studies in mice (Du et al., 2010; Zhao et al., 2013; Ding et al., 2016) and was chosen to avoid possible adverse effects. In our recent study (Liu et al., 2014), mice seemed to be well tolerated with daily treatment with the same dose for 28 days. Paeonol has a minimal systemic toxicity (LD_50_ = 3430 mg/kg) when orally administered to mice (Zhang et al., 1996). The *in vivo* dose of paeonol we used is far less than the LD_50_ dose; thus, we did not evaluate the toxicity of this drug. In our *in vitro* study, the concentration of paeonol (0.4 mM) did not produce any notable cytotoxicity. This concentration of paeonol was found to have no effect on the viability of lung epithelial cells (Liu et al., 2014) and macrophages (Ping et al., 2014). The Chinese herb *P. suffruticosa* has therapeutic effects, such as sedation, hypnosis, antipyresis, analgesic, anti-inflammation, and immunoregulation in human (Zhang et al., 1996). Although extensive studies have been conducted to investigate the beneficial effects of paeonol in several animal models of diseases, investigation on its clinical use as a single compound appears to be limited.

In summary, our findings suggest a novel role for paeonol regarding the alleviation of oxidative stress, inflammation, and fibrosis in the lungs induced by bleomycin *in vivo* and the suppression of the TGF-β1-induced fibrotic responses *in vitro* by inhibiting MAPKs/Smad3 signaling. While several drugs have been proposed for the treatment of pulmonary fibrosis (Moeller et al., 2008), our findings support the notion that paeonol can potentially be used as a treatment for this disease.

## Author contributions

ML, AL, HK and DP conducted the studies, analyzed the data and interpreted the data. ML and AL wrote the paper. TL and YK led the project, interpreted the data and wrote the paper.

### Conflict of interest statement

The authors declare that the research was conducted in the absence of any commercial or financial relationships that could be construed as a potential conflict of interest.
