# Supplementary material for: Prevention of Bleomycin-Induced Pulmonary Inflammation and Fibrosis in Mice by Paeonol
Source: Front Physiol. 2017 Mar 31;8:193. doi: 10.3389/fphys.2017.00193 (PMC5374202; doi:10.3389/fphys.2017.00193)
Supplement: Supplementary file 1 [file Presentation1.PDF]

Prevention of Bleomycin-Induced Pulmonary Inflammation and Fibrosis in Mice by Paeonol

Meng-Han Liu[1], An-Hsuan Lin[1], Hsin-Kuo Ko[2], Diahn-Warng Perng[2], Tzong-Shyuan Lee[1,*] and Yu Ru Kou[1,*]

Supplemental Data

Human lung fibroblasts were exposed to 1 μg/ml bleomycin for 24 hours, a dose that was suggested by a previous study (Am J Pathol. 165: 659-669, 2004). As shown in figure S1, this exposure of bleomycin did not significantly alter the expression of α-smooth muscle actin (α-SMA) and type 1α1 collagen (COL1A1). A combined treatment with paeonol treatment also did not affect the expression of these two fibrotic markers.

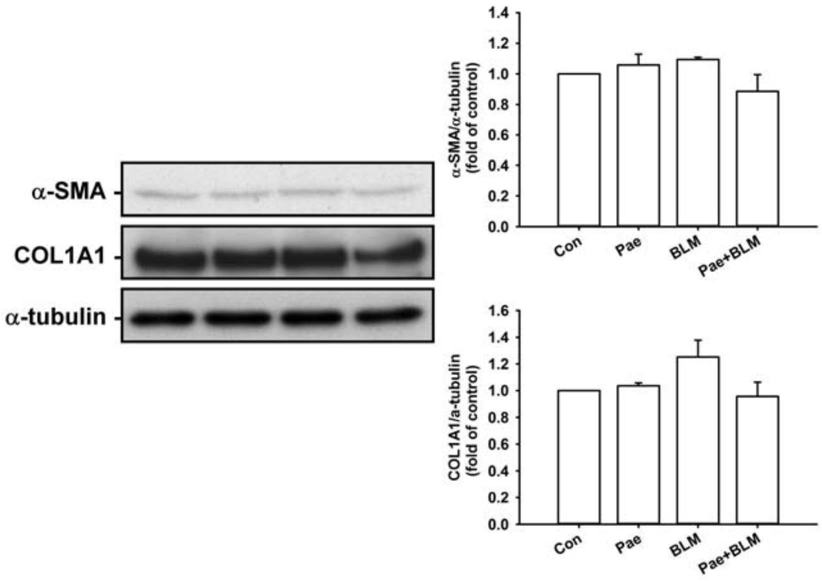

FIGURE S1 │ Exposure to bleomycin (BLM) does not alter expression of α-smooth muscle actin (α-SMA) and type 1α1 collagen (COL1A1) in human lung fibroblasts. (A) Representative images of Western blot results: (B and C) Quantitative data of expression. Protein samples were harvested 24 hours after BLM treatments and data were measured by Western blot analysis. Paeonol (Pae; 0.4 mM pretreatment was made 1 hour prior to BLM exposure. Data in each group are mean ± SEM from 4 independent experiments. No significance was detected between any two groups.
